# Supplementary material for: Multiplex Screening for Interacting Compounds in Paediatric Acute Myeloid Leukaemia
Source: Int J Mol Sci. 2021 Sep 21;22(18):10163. doi: 10.3390/ijms221810163 (PMC8468645; doi:10.3390/ijms221810163)
Supplement: Supplementary file 1 [file ijms-22-10163-s001.zip › ijms-1334665-supplementary.pdf]

A

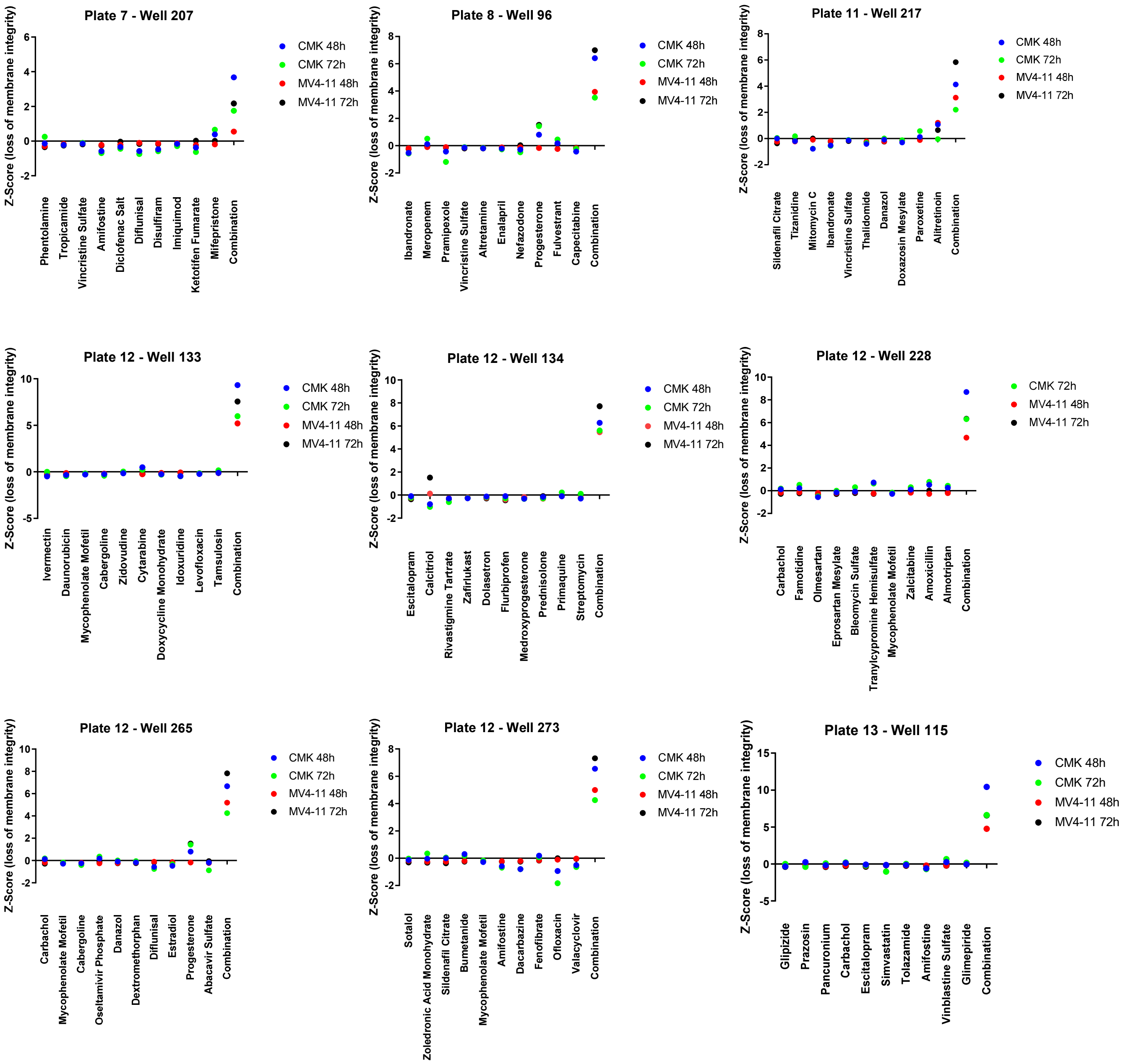


B

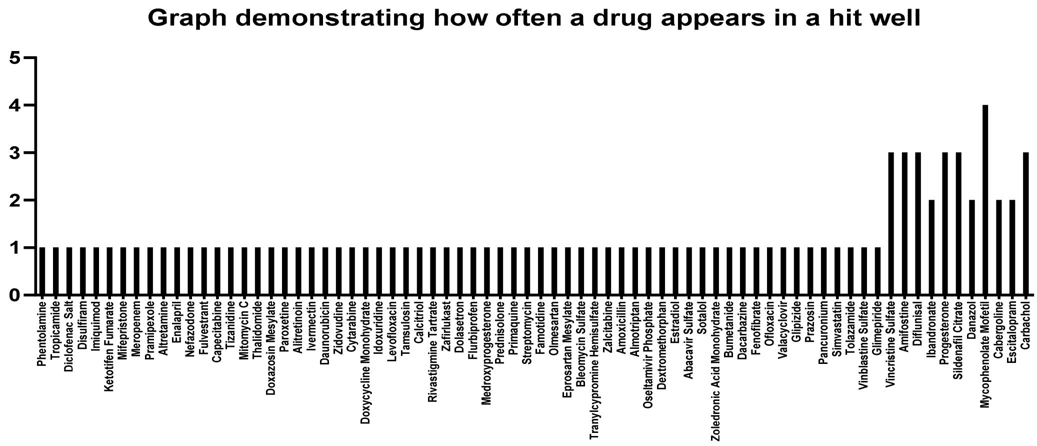


***Supplementary Figure S1:*** *Successful combinations from the MuSICAL combination screen*. (A) 9 combinations overlapped both CMK and MV4-11 cell lines and demonstrated a Z-Score>2 while each of the single agents demonstrated a Z-Score <2. Plate 13 – well 115 was taken forward for deconvolution. (B) A graph demonstrating the number of times each drug appeared in a successful combination.

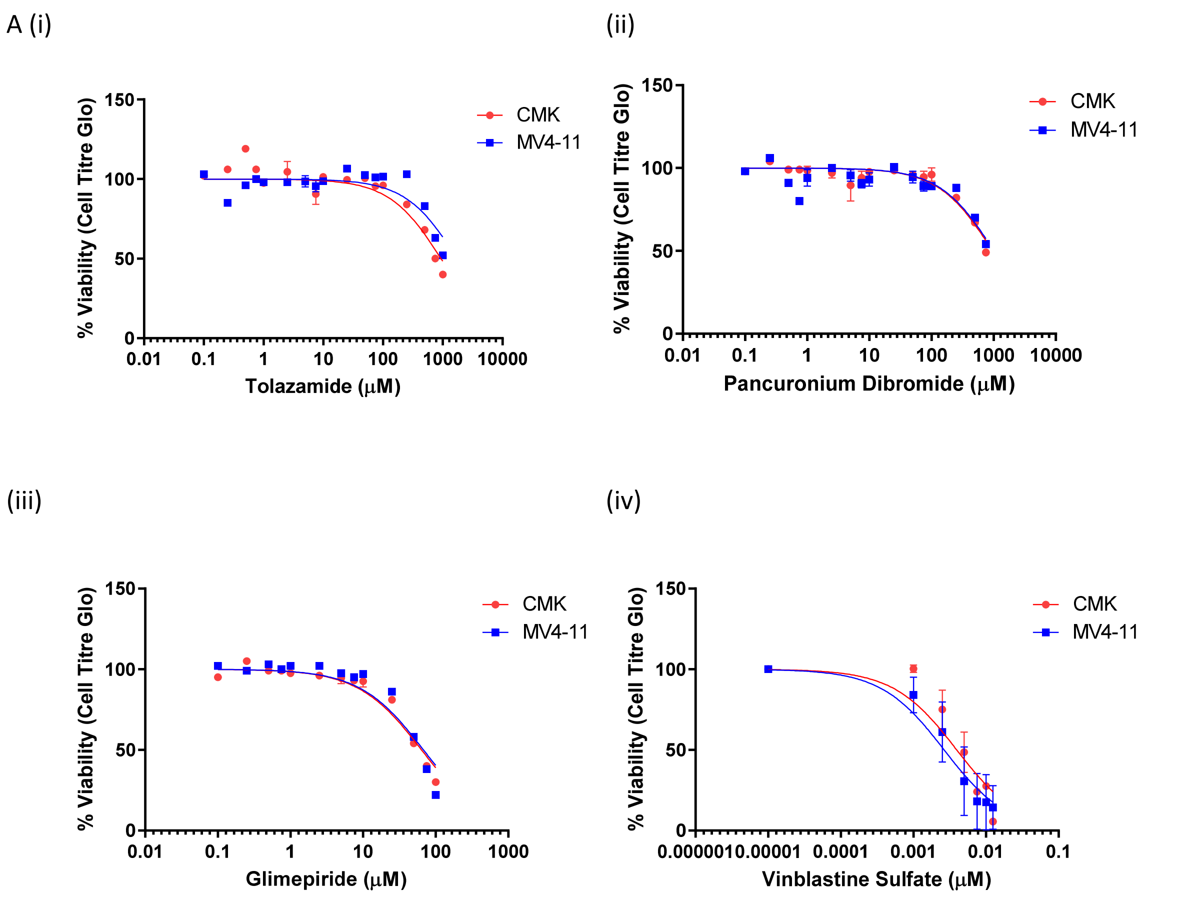


**Supplementary Table S1** – *Approximate IC50( µM) concentrations for CMK and MV4-11 cell lines following treatment with increasing concentrations of Tolazamide, Pancuronium Dibromide, Glimepiride and Vinblastine Sulfate*

| Drug Name | CMK (~IC50µM) | MV4-11 (~IC50µM) |
|---|---|---|
| **Tolazamide** | 937.2 | 1748 |
| **Pancuronium Dibromide** | 961.2 | 1007 |
| **Glimepiride** | 63.84 | 67.87 |
| **Vinblastine Sulfate** | 0.003952 | 0.002648 |

***Supplementary Figure S2:*** Dose response curves for paediatric AML cell lines following treatment with increasing concentrations of Tolazamide, Pancuronium, Glimepiride and Vinblastine Sulfate.

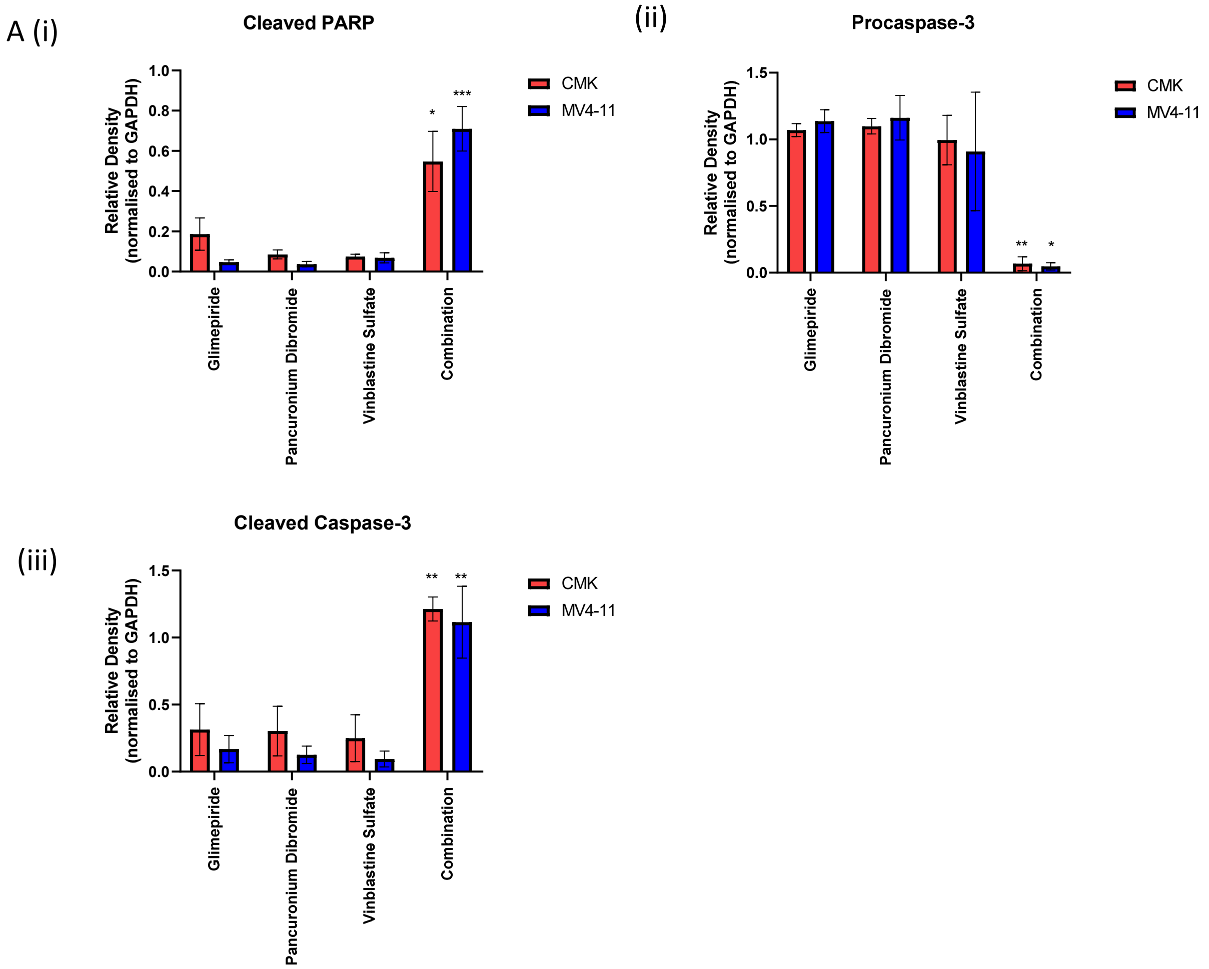


***Supplementary Figure S3:*** Densitometry analysis of (i) cleaved PARP, (ii) pro-caspase-3 and (iii) cleaved caspase-3 for CMK and MV4-11 cell lines treated with Glimepiride, Pancuronium Dibromide and Vinblastine Sulfate as single agents and as a triple combination. Results are representative of three independent experiments. **= P <0.05, **=P <0.01, ***P= <0.001.*

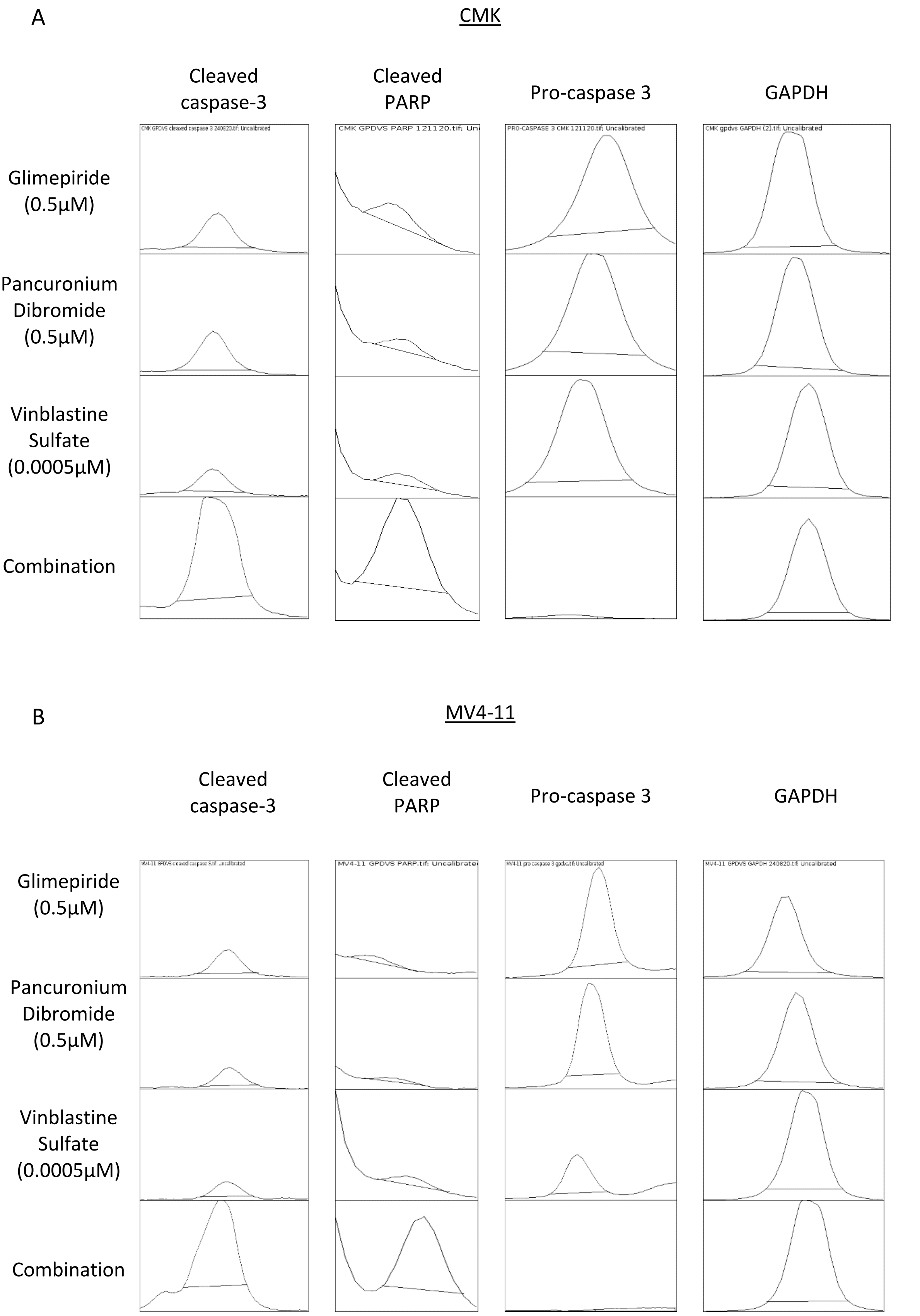


***Supplementary Figure S4:*** Representative histograms from densitometry analysis. (A) CMK (B) MV4-11.

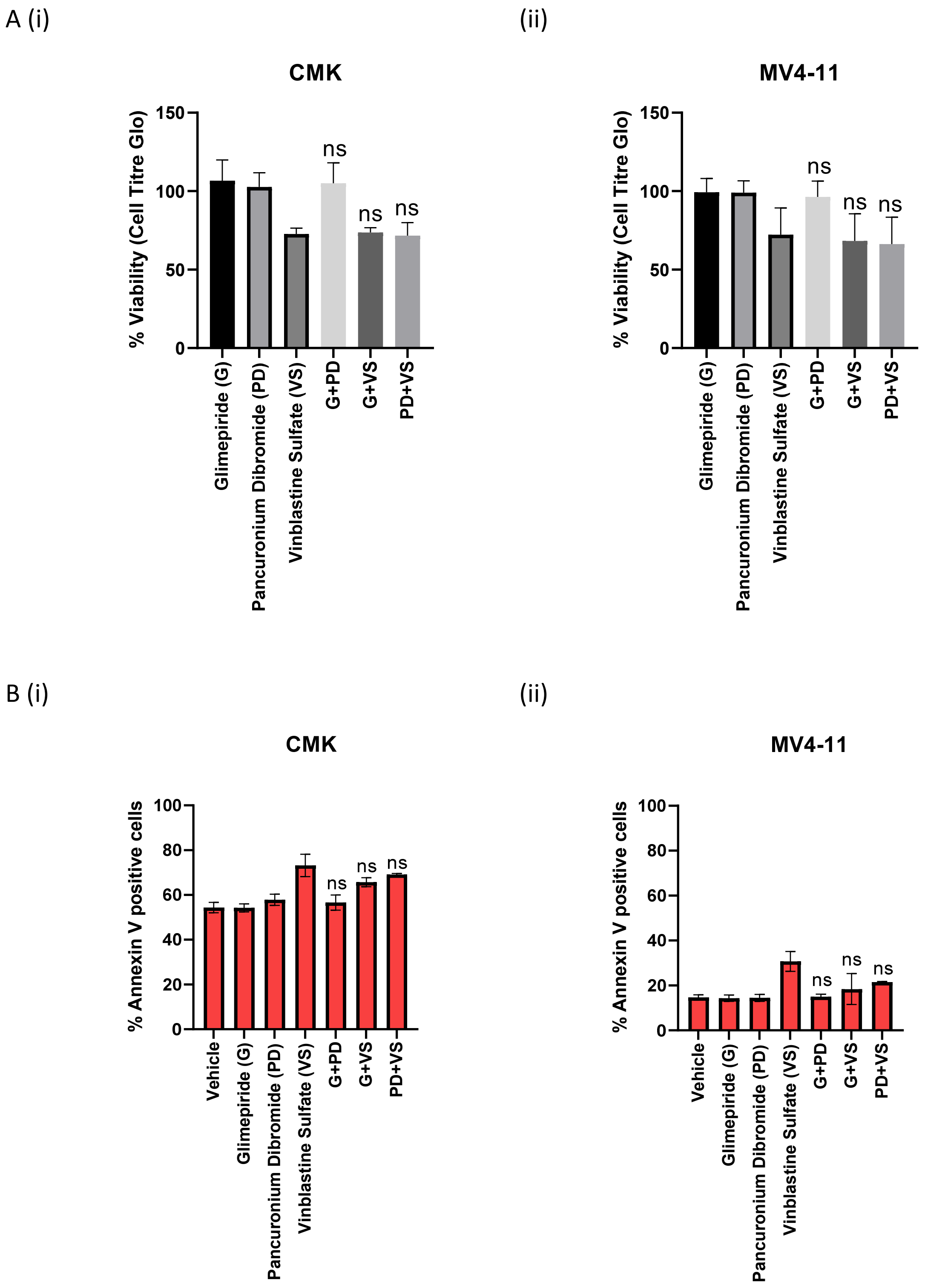


***Supplementary Figure S5:*** *Investigating the effects of the pairwise combinations of Glimepiride, Pancuronium Dibromide and Vinblastine Sulfate.* (A) CellTiter-Glo® luminescence assay was used to investgate the cell viability of (i) CMK and (ii) MV4-11 cell lines following 72h treatment with Glimepiride (0.5µM), Pancuronium Dibromide (0.5µM) and Vinblastine Sulfate (0.0005µM) as single agents and as pairwise combinations. (B) Flow cytometry analysis of Annexin V/PI positive cell population of (i) CMK and (ii) MV4-11 cell lines following 72h treatment with Glimepiride (0.5µM), Pancuronium Dibromide (0.5µM) and Vinblastine Sulfate (0.0005µM) as single agents and as pairwise combinations. *ns = non-significant.*

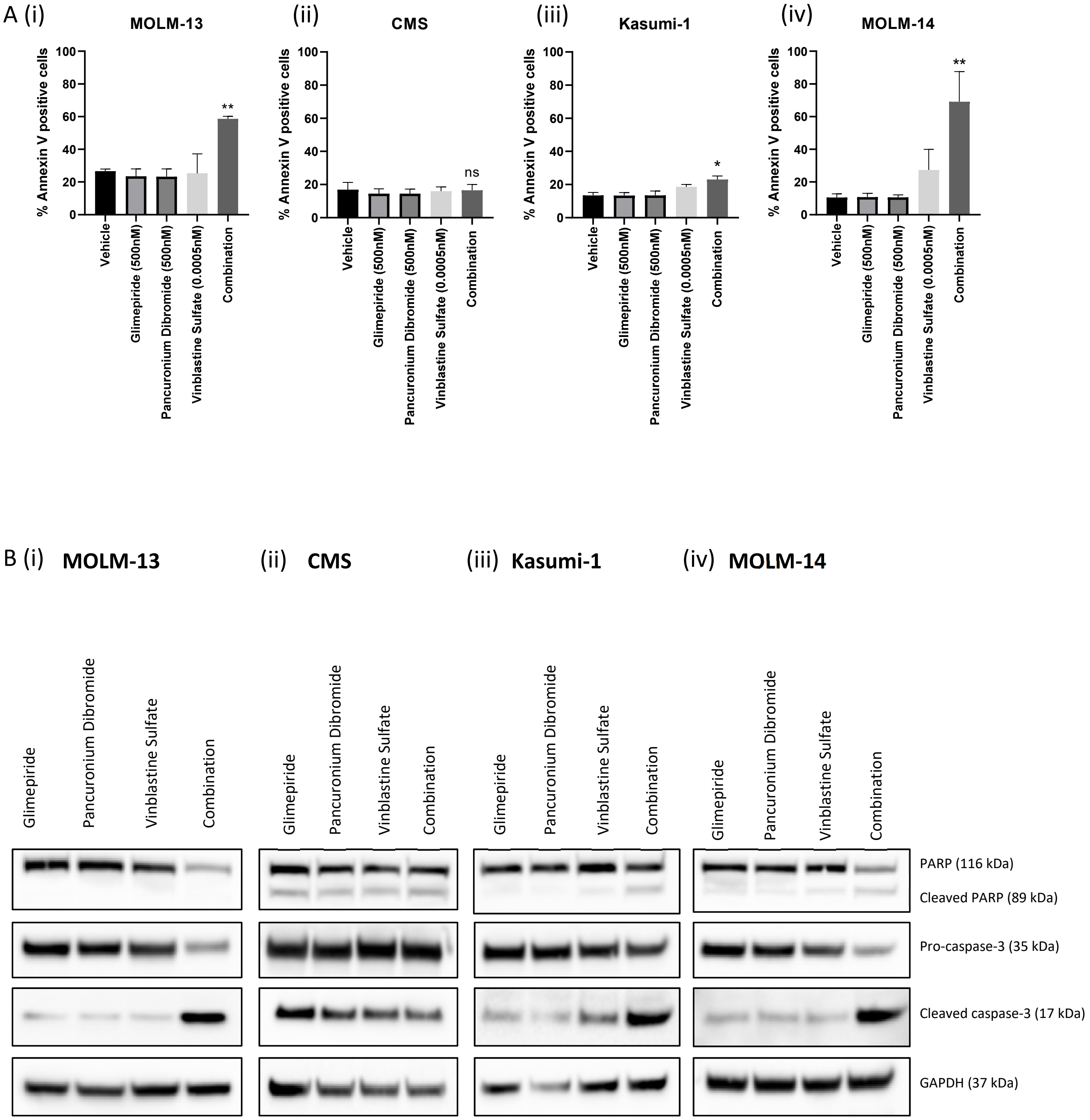


***Supplementary Figure S6:*** *Investigating the induction of apoptosis following treatment with glimepride, pancuronium dibromide and vinblastine sulfate as a triple combination across multiple pediatric AML cell lines.* (A) Flow cytometry analysis of Annexin V/PI positive cell population of (i) MOLM-13, (ii) CMS, (iii) Kasumi-1 and (iv) MOLM-14 cell lines following 72h treatment with Glimepiride (0.5μM), Pancuronium Dibromide (0.5μM) and Vinblastine Sulfate (0.0005μM) as single agents and as a triple combination. (B) Western blot analysis of PARP, Pro-Caspase-3, and cleaved Caspase-3 in the (i) MOLM-13, (ii) CMS, (iii) Kasumi-1 and (iv) MOLM-14 cell lines following 72h treatment with Glimepiride (0.5μM), Pancuronium Dibromide (0.5μM) and Vinblastine Sulfate (0.0005μM) as single agents and as a combination. GAPDH was used as a loading control. Results shown are representative of three independent experiments. *ns = non-significant*, **= P <0.05, **=P <0.01.*
